# A comprehensive investigation of the anion inhibition profile of a β-carbonic anhydrase from *Acinetobacter baumannii* for crafting innovative antimicrobial treatments

**DOI:** 10.1080/14756366.2024.2372731

**Published:** 2024-07-16

**Authors:** Viviana De Luca, Simone Giovannuzzi, Claudiu T. Supuran, Clemente Capasso

**Affiliations:** a Department of Biology, Agriculture and Food Sciences, National Research Council (CNR), Institute of Biosciences and Bioresources, Naples, Italy; b Neurofarba Department, Pharmaceutical and Nutraceutical Section, University of Florence, Sesto Fiorentino, Italy

**Keywords:** *Acinetobacter baumannii*, carbonic anhydrase, enzyme kinetic, anions, antibiotic resistance

## Abstract

This study refers to the intricate world of *Acinetobacter baumannii*, a resilient pathogenic bacterium notorious for its propensity at antibiotic resistance in nosocomial infections. Expanding upon previous findings that emphasised the bifunctional enzyme PaaY, revealing unexpected γ-carbonic anhydrase (CA) activity, our research focuses on a different class of CA identified within the *A. baumannii* genome, the β-CA, designated as 𝛽-AbauCA (also indicated as CanB), which plays a crucial role in the resistance mechanism mediated by AmpC beta-lactamase. Here, we cloned, expressed, and purified the recombinant 𝛽-AbauCA, unveiling its distinctive kinetic properties and inhibition profile with inorganic anions (classical CA inhibitors). The exploration of 𝛽-AbauCA not only enhances our understanding of the CA repertoire of *A. baumannii* but also establishes a foundation for targeted therapeutic interventions against this resilient pathogen, promising advancements in combating its adaptability and antibiotic resistance.

## Introduction

*Acinetobacter baumannii* is a pathogenic microorganism that is widely recognised for its involvement in both hospital-acquired and community-based infections, especially those related to ventilator use, such as ventilator-associated pneumonia^1–4^. The pathogen infamy is due to its exceptional genomic flexibility and the multitude of virulence factors it possesses, which greatly enhance its ability to cause infections^1^^,^^2^^,^^4^. *A. baumannii* lies in its remarkable ability to acquire novel antibiotic resistance determinants, coupled with its inherent tolerance to the diverse stresses prevalent in the hospital environment^5–7^. This adaptability facilitates the transmission of pathogens among patients and maintains the contamination of healthcare settings for extended durations^5^^,^^6^. The development of antimicrobial resistance in *A. baumannii* is a complicated and changing process that makes it very difficult to treat clinically, especially since multidrug resistance is becoming more common^8–11^. This includes resistance to carbapenems (imipenem, meropenem, and doripenem), a group of strong broad-spectrum antibiotics that are usually only used as a last resort to treat very sever bacterial infections^12–14^. The multifaceted nature of antimicrobial resistance in *A. baumannii* underscores the need for innovative and targeted therapeutic approaches to mitigate the growing clinical implications of this resilient pathogen^12^^,^^15^^,^^16^. The exploration of phage therapy as a viable alternative and adjunct to conventional antibiotics has become a critical research focus in the post-antibiotic era^17–20^. Despite their advantages such as cost-effectiveness, widespread availability, and high specificity, phage therapy has several limitations. The utilisation of phage-derived enzymes presents a promising avenue for combating “superbugs”; however, their applicability and safety for clinical use require thorough research^21–24^. In addition to phage therapy, other approaches such as active immunisations, passive immunisations, and antimicrobial peptides (AMPs) have emerged as potential strategies to address the challenges posed by antibiotic-resistant bacteria. Active immunisation involves stimulating the body’s immune system to produce its own protective response against specific pathogens, typically through the administration of vaccines^25^. Conversely, passive immunisation entails the direct introduction of pre-formed antibodies or immunoglobulins into the body to provide immediate, temporary protection. Both active and passive immunisations offer valuable tools in the fight against bacterial infections, complementing the efforts of phage therapy^26^^,^^27^^,^. Furthermore, antimicrobial peptides (AMPs), naturally occurring molecules with potent antimicrobial properties, have garnered attention for their ability to disrupt bacterial cell membranes and inhibit essential bacterial processes^28^. AMPs represent a promising avenue for therapeutic development due to their broad-spectrum activity and relatively low likelihood of inducing bacterial resistance^29^. Beyond these, innovative strategies such as CRISPR-based antimicrobial approaches, probiotics, and bacteriophage cocktail therapy are also being explored^30^. CRISPR-based antimicrobial approaches involve the use of CRISPR-Cas systems to target and destroy specific bacterial sequences, offering a precise and customisable means of combating antibiotic-resistant bacteria^30^. Probiotics, which involve the administration of beneficial bacteria to restore microbial balance in the body, hold potential for preventing and treating bacterial infections^31^. Bacteriophage cocktail therapy, similar to phage therapy, utilises a mixture of phages to target a broader range of bacterial strains, potentially enhancing treatment efficacy^32^. These diverse approaches enhance our understanding of the multifaceted strategies available for combating antibiotic-resistant bacteria and underscores the importance of continued research into alternative antimicrobial interventions.

In an intriguing exploration of the metabolic intricacies and stress response mechanisms of *A. baumannii*, the bifunctional enzyme PaaY takes centre stage^33^. This enzyme, encoded by the FQU82_01591 gene, is a key player in the degradation of toxic metabolites via the bacterial phenylacetic acid (PA) pathway^34^^,^^35^. It functions as a thioesterase, catalysing the hydrolysis of thioester bonds and significantly contributing to the detoxification process. A surprising discovery regarding PaaY function is that it has γ-carbonic anhydrase (CA, EC 4.2.1.1) activity, which makes it even more important in the context of microbes^36–38^. Bacterial carbonic anhydrases play a pivotal role in maintaining the crucial acid-base equilibrium and pH stability within bacterial cells^39–43^. They accelerate the reversible hydration of carbon dioxide to bicarbonate and protons, which is a fundamental reaction in carbon dioxide regulation and pH homeostasis^39^^,^^44^^,^^45^. Thus, the physiological importance of bacterial CAs encompasses various crucial aspects, such as metabolic functions, respiratory processes, acid-base equilibrium, and implications for virulence^46^^,^^47^. The X-ray crystal structure of PaaY with bicarbonate revealed a trimeric structure featuring a canonical γ-CA active site^36^. This structure also lays the groundwork for understanding thioesterase activity. This enzyme displays a penchant for lauroyl-CoA as its preferred substrate during thioesterase activity assays, introducing an element of specificity to its enzymatic repertoire. Furthermore, PaaY knockout is critical for biological processes in *A. baumannii*^36^. The outcomes encompass a restricted growth trajectory in media abundant in phenylacetic acid (PA), a reduction in the magnitude of biofilm formation, and an increased susceptibility to hydrogen peroxide^48^. Thus, within its biochemical complexity, PaaY transcends its role as a mere enzyme, emerging as a pivotal character shaping microbial destiny and leaving researchers and readers alike captivated by the biochemical revelations it brings to light. A recent comprehensive exploration of the *A. baumannii* genome undertaken by our research groups revealed the presence of 𝛼 and 𝛽 CAs in addition to 𝛾 CAs. In 2023, Colquhoun and co-workers have demonstrated that an insertion or deletion mutation in the *canb* gene, which encodes a 𝛽-CA (putative carbonic anhydrase [Acinetobacter baumannii ATCC 17978], results in a significant loss of viability when the adc-7 gene overexpress ADC-7 (AmpC beta-lactamase)^49^. Thus, CanB, the protein encoded by the *canb* gene, has a crucial role in providing essential bicarbonate ions in the initial phase of UTP synthesis for the formation of components such as UDP-N-acetylglucosamine and peptidoglycan (PG) within cellular processes, maintaining cell viability under conditions of ADC-7 overexpression^49^. This discovery deepens our curiosity about the CA-classes of *A. baumannii*. Specifically, it prompts us to synthesise and heterologously express the recombinant CanB, here designated as 𝛽-AbauCA, to gain insights on the CA repertoire of *A. baumannii*. The kinetic properties of 𝛽-AbauCA were elucidated and provided crucial insights into its catalytic efficiency. Furthermore, the investigation delved into the *in vitro* inhibition patterns of 𝛽-AbauCA, exploring its response to a diverse range of classical CA inhibitors (CAIs), such as inorganic metal-complexing compounds^50^^,^^51^. The orchestrated analysis of the kinetic constants of 𝛽-AbauCA, coupled with an exhaustive exploration of its reactivity to classical CAIs, epitomises a scholarly endeavour of profound significance. Beyond the immediate context of elucidating the CA repertoire of *A. baumannii*, this study holds promise for translational applications, thereby augmenting the prospects of therapeutic advancements.

## Materials and methods

### Chemicals and instruments

The reagents and equipment employed in this investigation were acquired from diverse suppliers. Isopropyl b-D-1-thiogalactopyranoside (IPTG) and antibiotics were procured from Merck (Darmstadt, Germany). The Affinity column (His-Trap FF) and molecular weight markers were sourced from Cytiva (Uppsala, Sweden). Furthermore, the AKTA Prime purification system, obtained from Cytiva, the SX20 Stopped-Flow instrument from Applied Photophysics (Leatherhead, UK), and the SDS–PAGE apparatus from BioRAD (Hercules, California, USA) were utilised. All other chemicals used were of reagent-grade quality. All of the recombinant enzymes utilised in this research were generated in-house, whereas the highest commercially available grade of salts and small molecules were obtained from Merck (Darmstadt, Germany).

### Gene identification, synthesis, cloning, and heterologous expression

The process of *A. baumannii CA* gene (*canb*, A1S_0984) identification, synthesis, and cloning involved multiple stages. Initially, the gene was identified from the genomic database NCBI and custom-designed by GeneArt Company (Thermo Fisher Scientific, Milan, Italy). Subsequently, it was cloned into the expression vector pET100/D-TOPO (Invitrogen, Palo Alto, CA, USA), resulting in the plasmid pET100D-Topo/𝛽-AbauCA. *Escherichia coli* BL21 (DE3) cells (Agilent) were transformed with the pET100D-Topo/𝛽-AbauCA vector, and induction with IPTG (1 mM) led to the overexpression of the recombinant 𝛽-AbauCA. A supplement of 0.5 mM ZnCl_2_ was introduced into the colture to facilitate proper protein folding. After 5 h from the IPTG induction, cells were harvested, and sonication was employed for cellular disruption. 𝛽-AbauCA, produced as a His-tag fusion protein, was purified using a nickel affinity column (His-Trap FF) connected to an AKTA Prime system. Elution was achieved with a specific buffer composition as previously described^52^. The recovered 𝛽-AbauCA exhibited a purity of 95%. Protein quantification was performed using the Bradford method by BioRAD^53^. A 12% SDS-PAGE and Coomassie Brilliant Blue-R staining were employed for analysis^54^. Protonography on the SDS-PAGE gel was conducted to detect hydratase activity, following the method outlined by Capasso and colleagues^55^. The CA activity assay, based on CO_2_ conversion to bicarbonate, was conducted at 0 °C, and Wilbur-Anderson units were calculated to determine enzyme activity. For the Western-Blot, the 𝛽-AbauCA transferred to a PVDF membrane using a Trans-Blot SD Cell (Bio-Rad, Hercules, CA, USA) and transfer buffer containing 25 mM Tris, 192 mM glycine, and 20% methanol. To conduct the His-Tag Western blot, the Pierce Fast Western Blot Kit (Thermo Scientific, Waltham, MA, USA) was employed following the procedure reported in the manual. Briefly, the blotted membrane was submerged in Fast Western 1 Wash Buffer to remove residual transfer buffer. Subsequently, the Primary Antibody Working Dilution was applied to the membrane, and incubation ensued for 30 min at RT (room temperature) with periodic shaking. Following this, the blot was withdrawn from the primary antibody solution and incubated with Fast Western Optimised HRP Reagent Working Dilution for 10 min at room temperature. The membrane underwent two washes with approximately 20 ml of Fast Western 1 Wash Buffer. Finally, the blot was then incubated with Detection Reagent Working Solution for 1–5 min at room temperature. Then, the excess of liquid was removed, and the blot exposed to the Invitrogen iBright CL1500 Imaging System. This meticulous process ensured the precise visualisation and detection of the His-tagged 𝛽-AbauCA on the PVDF membrane.

### Exploration of enzymatic reaction rate and inhibition profile with inorganic anions

The kinetic parameters of the 𝛽-AbauCA catalysed CO_2_ hydration reaction were investigated using an Applied Photophysics stopped-flow instrument^56^^,^^57^ using the conditions described in earlier investigations from our groups^58^. Pre-incubation of inhibitor and enzyme solutions for 15 min at room temperature preceded the assay. Inhibition constants were calculated using the Cheng-Prusoff equation^59^^,^^60^. These values represent averages from at least three separate determinations.

### Phylogenetic analysis

Phylogenetic analysis was conducted to elucidate the evolutionary relationships among the amino acid sequences of interest. Multiple sequence alignment was achieved using the MUSCLE 3.1 software, which employs advanced algorithms to generate accurate alignments of protein sequences^61^. This step facilitated the identification of conserved regions and variations within the sequences, crucial for inferring evolutionary connections. The dendrogram was generated employing PhyML 3.0 software, which employs a maximum likelihood approach to determine the tree topology that best fits the observed data^62^.

## Results and discussion

### Exploring the primary structure and phylogenetic analysis of 𝛽-AbauCA

**Figure 1.**
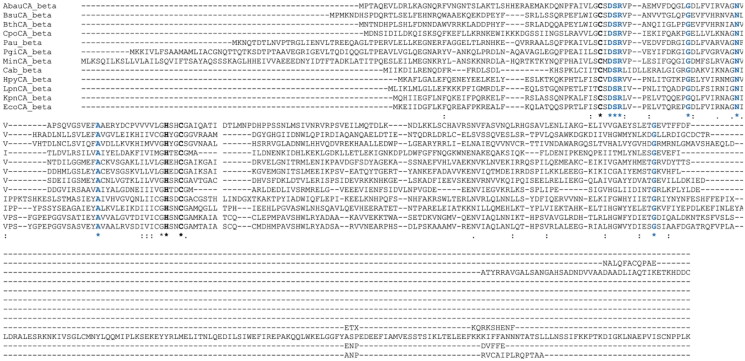
Multiple sequence alignment of β-CA amino acid sequences. This alignment was conducted using Clustal X and β-CA from the bacterial species listed in Table 1. This representation highlights in black bold the conserved zinc ligands: two cysteines (Cys) and one histidine (His). Light blue indicates residues that are completely conserved across all sequences. The symbol (∗) denotes the positions where there is an identity across all the aligned sequences. The symbol (:) signifies conserved substitutions and (.) indicates semi-conserved substitutions, reflecting variations that are not as stringent as complete conservation but still show some level of similarity or conservation.

The elucidation of the primary sequence of the 𝛽-AbauCA from *A. baumannii* was effectively accomplished through the exploration of the genomic databases hosted by the National Centre for Biotechnology Information (NCBI)^63^. 𝛽-AbauCA was meticulously identified with the accession number GenBank: ABO11416.2 (204 amino acid residues; genome locus_tag = A1S_0984). After acquiring the genetic sequence, it underwent a meticulous investigation using Muscle 3.5, a program known for its robust sequence alignment capabilities^64^. This comprehensive analysis involved a detailed inspection to ensure the accuracy of 𝛽-AbauCA sequence and its alignment with other recognised bacterial 𝛽-CAs (Figure 1).

In line with the analysis of other β-CAs investigated to date, the preservation of residues Cys, His, and Cys (highlighted in bold black in Figure 1) across all the amino acid sequences of bacterial β-CAs under consideration emphasises their crucial role as zinc ligands within the active site. The arrangement of the Zn^2+^ ion in the active site has been clarified by insights obtained from previously acquired X-ray crystal structures^65^. These three conserved residues serve as key players in coordinating the zinc ion, stabilising the active site, and contributing to the enzyme catalytic process^66^. Furthermore, the catalytic process involves the participation of a deprotonated water molecule, serving as the fourth ligand^67–69^. This water molecule acts as the pivotal Zn^2+^-hydroxide species, facilitating the catalytic attack on carbon dioxide (CO_2_)^70^. The coordination of these residues as well as the water molecule form a molecular ensemble that orchestrates the β-CA enzymatic activity. Additionally, Figure 1 reveals a distinct pattern of highly conserved residues, highlighted in blue, further underscoring the importance of these amino acids in the structure and function of β-CAs across different bacterial species. These residues play a dual role by contributing not only to the stability of the enzyme but also to its catalytic efficiency in mediating the vital conversion of CO_2_ to bicarbonate.

**Figure 2.**
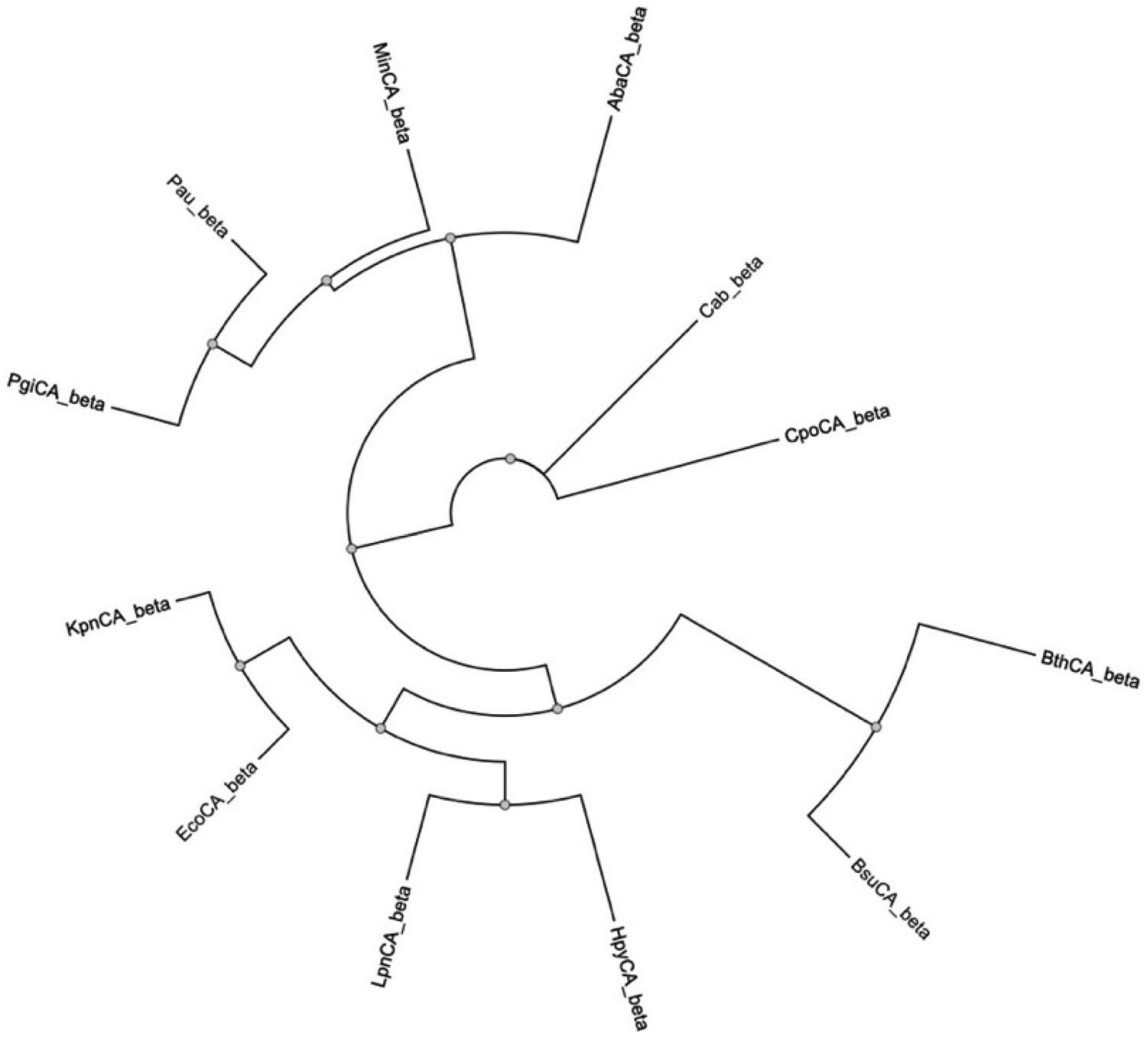
Phylogenetic analysis of 𝛽-AbauCA and other 𝛽-CAs from different bacterial species. The phylogenetic tree was constructed using PhyML 3.0.

The integration of primary structure analysis and phylogenetic analysis is a powerful approach for unravelling the complexities of β-CAs^71^. It provides a holistic view of the molecular, structural, and evolutionary aspects of these enzymes, facilitating a more nuanced understanding of their functions and adaptations^71^^,^^72^. Thus, in this context, a phylogenetic tree has been constructed using all the amino acid sequences listed in Table 1 (Figure 2)^62^.

**Table 1.** The following table includes the β-CA sequences obtained from bacterial species and utilised in both the multiple sequence alignment and phylogenetic analyses.

Name	Acronym	Accession number
*Klebsiella pneumoniae*	KpnCA_beta	WP_002907811.1
*Acinetobacter baumannii*	AbaCA_beta (𝛽-AbauCA or CanB)	ABO11416.2
*Brucella suis*	BsuCA_beta	AAN33967.1
*Burkholderia thailandensis*	BthCA_beta	WP_009893276.1
*Myroides injenensis*	MinCA_beta	WP_010254382.1
*Legionella pneumophila*	LpnCA_beta	WP_011946835.1
*Pseudomonas aeruginosa*	Pau_beta	WP_235375492.1
*Porphyromonas gingivalis*	PgiCA_beta	YP_001929649.1
*Escherichia coli*	EcoCA_beta	WP_047081292.1
*Methanobacterium thermoautotrophicum*	Cab_beta	1G5C_A
*Helicobacter pylori*	HpyCA_beta	YP_005769368.1
*Candidatus prometheoarchaeum*	CpoCA_beta	WP_147661847.1

In Figure 2, a conspicuous pattern emerges, revealing a distinctive association between 𝛽-AbauCA and 𝛽-CAs found in *Porphyromonas gingivalis*, *Pseudomonas aeruginosa*, and *Myroides injenensis*. This observation is particularly intriguing given that, excluding *M. injenensis*, our research groups have extensively investigated the inhibition profiles using various classical CA Inhibitors (CAIs), such as sulphonamides and anions, targeting the 𝛽-CAs encoded by the genomes of *P. gingivalis*^73^^,^^74^ and *P. aeruginosa*^75–77^. The practical implications of these CAIs were effectively demonstrated in *P. aeruginosa*, showcasing their ability to reduce calcium deposition^75^. This reduction, which influences crucial bacterial cellular processes including biofilm formation and virulence, underscored the potential utility of such inhibitors in combatting microbial pathogenicity. Turning the focus to the β-CA from *P. gingivalis*, the CAIs presented promising avenues for breakthroughs in eradicating this pathogenic organism^73^^,^^74^.

Thus, considering the remarkable effectiveness of these inhibitors against the β-CAs from *P. gingivalis* and *P. aeruginosa*, it becomes plausible to explore their potential application against the β-CA of *A. baumannii*. This avenue holds promise as a compelling alternative to conventional antibiotics, offering a potential strategy to counteract *A. baumannii* growth and virulence within the host.

### Heterologous overexpression and active nature of the purified recombinant 𝛽-AbauCA

**Figure 3.**
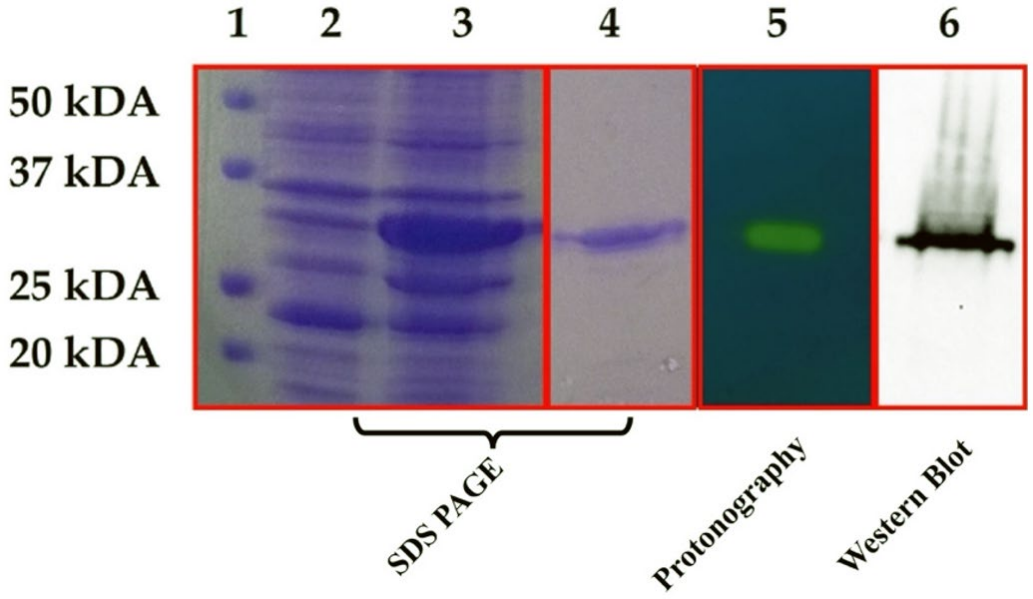
Examination of the purified recombinant 𝛽-AbauCA through the use of three analytical techniques, specifically SDS-PAGE, Western blot, and Protonography. The results obtained from SDS-PAGE and Western blotting provided information on the protein’s molecular weight and identity, while Protonography revealed its enzymatic activity, which was visually represented by a distinct yellow band. The figure includes a legend that identifies the different lanes and their corresponding content. Lane 1 shows the molecular markers, ranging from 20 to 50 kDa (BioRad, Precision plus Protein Standards Dual Colour, catalog number: 161–0394), while lanes 2 and 3 display the cytoplasmic fraction from whole *E. coli* lysates before and after IPTG induction, respectively. Lane 4 represents the purified CA from the affinity column, and Lane 5 shows the activity of the 𝛽-AbauCA with an apparent molecular weight of 29.0 kDa. Finally, Lane 6 identifies the 𝛽-AbauCA band using an anti-His-Tag antibody.

In this investigation, recombinant DNA technology was employed to synthesise and clone the 𝛽-AbauCA gene. The engineered 𝛽-AbauCA, featuring a His-Tag tail comprising six histidine residues at its N-terminus, was heterologously overexpress in *Escherichia coli* resulting in the successful production of the desired cytoplasmic protein (Figure 3).

The cellular extract obtained post-sonication and centrifugation, ensured a highly effective recovery (5 mg) of 𝛽-AbauCA through the utilisation of an affinity column, specifically the His-select HF Nickel affinity gel (Figure 3, lane 4). The success of this purification was validated through SDS-PAGE and Western Blot analysis, revealing a subunit with an apparent molecular weight of approximately 29,000 Da, closely matching the theoretically calculated molecular weight of 27817.57 Da of the recombinant 𝛽-AbauCA as fusion protein (Figure 3). The protein showed a high degree of purity, too (Figure 3, lane 4). Following purification, the 𝛽-AbauCA catalytic activity was evaluate by protonography (Figure 3, lane 5). This innovative approach allows for a detailed analysis and visualisation of the catalytic function of CAs directly on the gel^78–80^. The resulting protonogram, illustrated in Figure 3, showcases a distinct yellow band, serving as a tangible indicator of ion production (H^+^) during the CO_2_ hydration reaction catalysed by 𝛽-AbauCA. This evidence unequivocally demonstrates the active nature of the produced recombinant 𝛽-AbauCA.

### Catalytic behavior of 𝛽-AbauCA

The ensuing analysis involved the determination of kinetic constants for 𝛽-AbauCA, providing insights into its catalytic behaviour. The following kinetic parameters were measured for this new enzyme for the CO_2_ hydration reaction at 25 °C and pH 8.3: k_cat_: 4.53 × 10^5^ s^−1^, Km: 15.4 mM, and k_cat_/Km of 2.94 × 10^7^ M^−1^×s^−1^, which are similar to corresponding data measured for another β-class enzyme, form the bacterium *Porphyromonas gingivalis* β-CA (k*_cat_* of 2.8 × 10^5^ s^−1^ and a k_cat_/K_m_ of 1.5 × 10^7^ M^−1^ × s ^−1^)^43^^,^^47^. Thus, 𝛽-AbauCA shows a significant, moderate catalytic activity for the physiological reaction, which is in the same range as that of the abundant human isoform hCA I and many bacterial CAs investigated earlier^43–47^.

### Inhibition patterns with metal-complexing anions

Anions, which are negatively charged ions, are integral to numerous physiological processes in microbial cells, including enzymatic reactions, membrane transport, and regulation of cellular pH^50^. CAs are enzymes that play a pivotal role in regulating intracellular pH by catalysing the reversible hydration of CO_2_ to bicarbonate and a proton^50^. By investigating how anions interact with 𝛽-AbauCA, researchers can gain insights into the intricate regulatory mechanisms governing microbial physiology and metabolism. Understanding these interactions can provide clues about how *A. baumannii* adapts to different environmental conditions and how it may develop resistance to antibiotics. Additionally, deciphering the structural catalytic interaction of these inhibitors with the enzyme’s catalytic pocket is fundamental for designing new and more potent inhibitors^50^. Thus, the inhibition patterns of 𝛽-AbauCA were explored in the presence of inorganic metal-complexing compounds. This multifaceted approach aims to unravel the enzymatic intricacies of 𝛽-AbauCA, contributing to a deeper understanding of its functional attributes and offering potential avenues for targeted drug design with the aim to offer advanced knowledges and putative therapeutic possibilities.

The following may be observed regarding β-AbauCA inhibition with anions and small molecules:

- Anions which showed poor inhibitory effects (Ki > 100 mM) were the halides, azide, bicarbonate, carbonate, perchlorate, tetrafluoroborate, hexafluorophosphate and triflate. Whereas the last 4 anions mentioned above show poor metal ions coordinating ability^50^^,^^51^^,^, the halides, azide and bicarbonate on the other had do bind effectively many metal ions, in solution and within enzyme active sites, and thus, these data are of interest and are difficult to be rationalised. For example, for the β-CA from *P gingivalis*, all halides were inhibitory in the low millimolar range (Table 2).
- Medium potency inhibition was observed with nitrate, nitrite, bisulphite, sulphate, selenate, pyrophosphate, fluorosulfonate and phenylarsonic acid, which showed Ki values in the range of 18.4–79.3 mM.
- Effective inhibition, in the low millimolar range was recorded for the following anions/small molecules: thiocyanate, cyanide, stannate, tellurate, divanadate, tetraborate, perrhenate, peroxydisulfate, trithiocarbonate, diethyldithiocarbamate, and sulfamide, which had Kis in the range of 1.9–8.2 mM (Table 2).
- The most effective β-AbauCA inhibitors were cyanate, hydrogensulfide, perrhuthenate, selenocyanide, iminodisulfonate, sulphamic acid and phenylboronic acid, with Kis of 0.29–0.96 mM.

**Table 2.** Inhibition of bacterial enzyme from *Porphyromonas gingivalis* (PgiCAβ) and *Acinetobacter baumannii* β-CA (AcbCAβ) with anions, measured by using a CO_2_ hydrase, stopped-flow assay.

	Ki (mM)^a^	
Anion	PgiCAβ^b^	𝛽-AbauCA^c^
F^-^	7.8	>100
Cl^-^	7.5	>100
Br^-^	15.9	>100
I^-^	21.4	>100
CNO^-^	0.76	0.79
SCN^-^	1.9	1.9
CN^-^	5.4	4.6
N_3_^-^	>100	>100
HCO_3_^-^	7.3	>100
CO_3_^2-^	3.7	>100
NO_3_^-^	>100	47.9
NO_2_^-^	7.8	31.1
HS^-^	4.5	0.40
HSO_3_^-^	>100	59.1
SO_4_^2-^	>100	79.3
SnO_3_^2-^	1.5	2.4
SeO_4_^2-^	9.2	18.4
TeO_4_^2-^	3.9	6.0
P_2_O_7_^4-^	8.2	33.6
V_2_O_7_^4-^	8.1	5.8
B_4_O_7_^2-^	7.2	6.9
ReO_4_^-^	2.3	8.1
RuO_4_^-^	3.2	0.96
S_2_O_8_^2-^	9.2	3.8
SeCN^-^	2.4	0.71
CS_3_^2-^	4.3	3.3
Et_2_NCS_2_^-^	0.23	8.2
ClO_4_^-^	>100	>100
BF_4_^-^	>100	>100
FSO_3_^-^	3.9	29.3
PF_6_^-^	8.2	>100
CF_3_SO_3_^-^	8.5	>100
NH(SO_3_)_2_^2-^	2.1	0.93
H_2_NSO_2_NH_2_	0.078	2.1
H_2_NSO_3_H	0.060	0.63
Ph-B(OH)_2_	0.077	0.29
Ph-AsO_3_H_2_	0.076	51.9

^a^Mean from 3 different assays, by a stopped-flow technique (errors were in the range of ± 5–10% of the reported values).

^b^From Ref.^47^

^c^This work.

## Conclusions

Colquhoun and co-workers demonstrated intricate interplay between CanB (here 𝛽-AbauCA) activity and β-lactamase overexpression revealing a potential mechanism for the observed lethality in *A. baumannii*^49^. The synthesis of peptidoglycan (PG), an essential component of the bacterial cell wall, relies on the availability of precursors, including uridine triphosphate (UTP)^49^^,^^81^. In this context, 𝛽-AbauCA plays a pivotal role by contributing to the synthesis of bicarbonate, a key precursor crucial for UTP production. Considering these critical findings, our study delved into the cloning, expression, and purification of 𝛽-AbauCA. The successful heterologous overexpression and purification of recombinant 𝛽-AbauCA allowed us to explore the enzyme kinetic properties, providing insights into its catalytic efficiency. The enzyme has a good catralytic activity for the CO_2_ hydration reaction, with kinetic parameters similar to those of hCA I and other bacterial enzymes characterised earlier. Moreover, our study extended its focus to investigate the inhibition profile of 𝛽-AbauCA, particularly with classical CAIs, such as inorganic anions^82^^,^^83^. Indeed, thiocyanate, cyanide, stannate, tellurate, divanadate, tetraborate, perrhenate, peroxydisulfate, trithiocarbonate, diethyldithiocarbamate, and sulfamide has low millimolar affinity for the enzyme, whereas the best inhibitors were cyanate, hydrogensulfide, perrhuthenate, selenocyanide, iminodisulfonate, sulphamic acid and phenylboronic acid, with submillimolar Ki values. This nuanced exploration aimed to unravel the enzyme’s response to various inhibitory agents, shedding light on potential strategies to modulate its activity. Understanding the inhibition profile of 𝛽-AbauCA contributes not only to the fundamental comprehension of *A. baumannii* physiology but also holds promise for the development of targeted therapeutic interventions.

## Data Availability

We will provide access to the data upon readers’ request.
